# Role of the inflammasome in chronic obstructive pulmonary disease (COPD)

**DOI:** 10.18632/oncotarget.17850

**Published:** 2017-05-13

**Authors:** Chiara Colarusso, Michela Terlizzi, Antonio Molino, Aldo Pinto, Rosalinda Sorrentino

**Affiliations:** ^1^ Department of Pharmacy, University of Salerno, ImmunePharma s.r.l., Fisciano, Salerno, Italy; ^2^ Department of Medicine and Surgery, Respiratory Division, University of Naples “Federico II”, Naples, Italy

**Keywords:** inflammation, COPD, lung injury, immune response, inflammasome, Pathology Section

## Abstract

Inflammation is central to the development of chronic obstructive pulmonary disease (COPD), a pulmonary disorder characterized by chronic bronchitis, chronic airway obstruction, emphysema, associated to progressive and irreversible decline of lung function. Emerging genetic and pharmacological evidence suggests that IL-1-like cytokines are highly detected in the sputum and broncho-alveolar lavage (BAL) of COPD patients, implying the involvement of the multiprotein complex inflammasome. So far, scientific evidence has focused on nucleotide-binding oligomerization domain-like receptors protein 3 (NLRP3) inflammasome, a specialized inflammatory signaling platform that governs the maturation and secretion of IL-1-like cytokines through the regulation of caspase-1-dependent proteolytic processing. Some studies revealed that it is involved during airway inflammation typical of COPD. Based on the influence of cigarette smoke in various respiratory diseases, including COPD, in this view we report its effects in inflammatory and immune responses in COPD mouse models and in human subjects affected by COPD. In sharp contrast to what reported on experimental and clinical studies, randomized clinical trials show that indirect inflammasome inhibitors did not have any beneficial effect in moderate to severe COPD patients.

## INTRODUCTION

Inflammation is a complex process which main activity is to remove dangerous stimuli to restore tissue homeostasis [1]. Although the physiological role of inflammation is to eliminate the cause/s of cell injury, an exaggerated or latent response could result in chronic and severe inflammation which can lead or contribute to the development of other diseases, as in the case of respiratory diseases. In this context, it is well-known that inflammation is one of the main feature of lung injuries and chronic respiratory diseases, such as chronic-obstructive pulmonary disease (COPD) [2].

**Figure 1:**
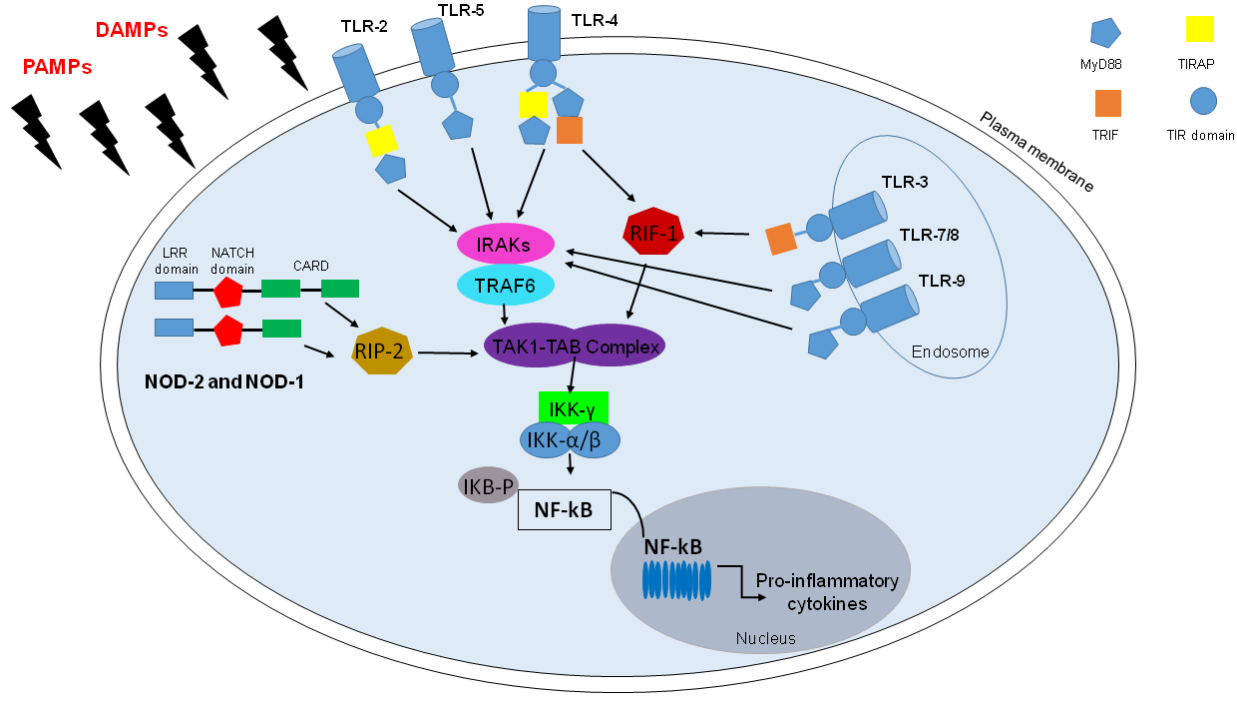
TLRs and NLRs in NF-kB activation TLRs and some NLRs, in particular, NOD1 and NOD2, activate nuclear factor (NF)-kB by recognizing PAMPs and DAMPs. TLRs are transmembrane receptors, except for TLR-3/7/8/9, with a leucine-rich repeats ((LRRs) motif), a Toll/IL-1 receptor (TIR) interaction domain required for forming multimers with TIR-containing adaptor molecules, namely myeloid differentiation primary response protein (MyD)88, MyD88 adaptor-like also known as TIR domain-containing adaptor protein, TIRAP), TIR domain-containing adaptor inducing IFN-b (TRIF). Upon stimulation, TLRs activate MyD88-dependent pathway: MyD88 recruits members of the IRAK family and TRAF6 for the activation of the TAK1/TAB complex, with the resultant IKK complex activation that triggers NF-kB and its translocation to the nucleus. However TLR-4, as TLR-3, active TRIF-dependent pathway which lead to NF-Kb activation with the recruitment of receptor-interacting protein-1 (RIP-1). Among NLRs there are NOD-1 and NOD-2 which present in the N-terminal portion Pyrin domain (PYD) or caspase-recruitment domain (CARD), a central NACTH domain and in the C-terminal portion a series of leucine-rich repeats (LRR). Though CARD-CARD interactions NOD-1 and NOD-2 recruit RIP-2 kinase that lead to TAK-1/TAB complex and then NF-kB activation.

Lung is a dynamic organ and it is highly susceptible to inhaled infectious agents and exogenous particulate matter (i.e. allergens, dusts, microorganisms) which can lead to infection, inflammation or tissue injury. Thus, a critical role in protecting the respiratory system from disease is played by the lining innate immune system that represents the first defense against pathogens and irritants [3]. In the last ten years several studies have demonstrated that both airway epithelial and mesothelial cells, together with innate and adaptive immune cells, are capable of triggering inflammatory events in response to endogenous and exogenous insults, following pattern recognition receptors (PRRs) triggering [2]. PRRs are expressed mainly on immune and inflammatory cells such as macrophages, monocytes, neutrophils and dendritic cells (DCs) and they recognize pathogen-associated molecular patterns (PAMPs) and damage-associated molecular patterns (DAMPs). Different types of PRRs include Toll-like receptors (TLRs), expressed either on the plasma membrane or in endosomal/lysosomal organelles [4], and nucleotide-binding oligomerization domain-like receptors (NLRs: i.e. NOD1 and NOD2) present within the cytoplasm (Figure 1) [5]. The stimulation of these receptors leads to signaling pathways that promote the release of inflammatory mediators due to the activation of NF-κB, a transcription factor, which is central in inflammation and immunity [6]. NF-κB and its activators IκB kinase (IKK) α/β play important roles in driving the inflammatory response by inducing the expression of pro-inflammatory and anti-apoptotic genes [7]. NF-κB activation in response to pro-inflammatory stimuli is regulated by IKK, which phosphorylates IκB and promotes its proteasome degradation and the release of NF-κB for nuclear translocation and gene transcription activation [6]. However, several reports have shown that NF-κB and IKKβ also influence anti-inflammatory responses, pointing at the resolution of acute inflammation (Figure1).

### COPD: an inflammatory disease

COPD is considered the fourth-leading causes of death worldwide, equally affecting females and males. It is predicted to become the fifth ranked cause of disability worldwide [8].

COPD is a disorder characterized by chronic bronchitis, mucus hypersecretion, chronic airway obstruction, airway remodeling, emphysema, shortness of breath, increased cough [9] with the consequent parenchymal destruction and loss of alveolar attachments [8], all symptoms that lead to progressive and irreversible decline of lung function [7]. Systemic effects are also observed in the skeletal muscle, heart and gut [7, 8, 9]. The decline of the clinical conditions of patients is exaggerated by exacerbation of the disease when bacterial or viral infections occur. However, the mechanism of COPD pathogenesis is yet poorly understood and the identification of novel therapeutic targets is clearly an unmet clinical need. Current therapies, i.e. long-acting bronchodilators and inhaled corticosteroids, are relatively efficient for COPD patients in that they are used to control the symptoms; however, there are still no drugs available that considerably reduce the progression/exacerbation of the disease and even worse, the mortality [10].

The classification of severity of COPD now includes four stages classified by spirometry: Stage I: Mild COPD characterized by mild airflow limitation; Stage II: Moderate COPD characterized by worsening airflow limitation; Stage III: Severe COPD characterized by further worsening of airflow limitation; Stage IV: Very Severe COPD characterized by severe airflow limitation plus the presence of chronic respiratory failure [11].

The development of COPD is caused by inhalation of noxious particles or gas, especially cigarette smoke (CS) that represents the first risk factor for this respiratory disorder [3]. It is believed that lung inflammation in COPD patients reflects the site of deposition of irritants from CS and particles of air pollution [12], which can cause chronic inflammation in a long-term manner.

### Inflammatory and Immune Responses in COPD

The inflammatory response in COPD involves both innate and adaptive immunity. However, structural cells, such as endothelial, airway and alveolar epithelial cells and fibroblasts [8], exposed to CS and/or air pollutants can trigger an inflammatory cascade resulting, for example, in the activation of macrophages and airway epithelial cells which in turn release cytokines and chemokines promoting the recruitment of other inflammatory cells (i.e. neutrophils, monocytes, lymphocytes) into the lungs [8-13]. To exacerbate the clinical outcome of COPD patients, CS and noxious environmental particles increase the susceptibility to infection/s. In this scenario, epithelial cells are activated and are involved in the release of inflammatory mediators, such as tumor necrosis factor (TNF-α), IL-1β, IL-6, granulocyte-macrophage colony-stimulating factor (GM-CSF) and IL-8 [14]. In particular, the percentage of macrophages recruited to the airways, lung parenchyma, bronchoalveolar lavage fluid (BALF) and sputum of patients with COPD, represents a grade of severity of this pulmonary disease [13]. COPD-associated macrophages, including alveolar macrophages, secrete inflammatory mediators, such as TNF-α, reactive oxygen species (ROS), proteolytic enzyme, such as metalloprotease and cathepsins, after cigarette smoke exposure [8-15]. As reported by Camarori et al. [16], most of the inflammatory proteins that are over-expressed in COPD macrophages are regulated by NF-κB which is active during the exacerbation phase. Macrophages in COPD derive from circulating monocytes, which migrate to the lungs in response to chemoattractants, such as CCL2 (also known as MCP1) which acts on CCR2, and CXCL1 which triggers CXCR2. There is increasing evidence that lung macrophages orchestrate COPD-associated inflammation through the release of chemokines that attract neutrophils, monocytes and T cells and the release of proteases, such as matrix metalloproteinase MMP-9 [17].

To note, an increase in activated neutrophils (CD16^high^ , CD54^+^, CD181/CXCR1^+^, A17^+^ cells), DCs [18] and CD8^+^ T cells, rather than CD4^+^ T cells, are found in sputum, BAL and airway smooth muscles of COPD patients. Airway neutrophilia in COPD is linked to mucus hypersecretion and to disease severity [8]*.* Neutrophils, recruited to the airways of COPD patients, secrete serine proteases, including neutrophil elastase (NE), cathepsin G, and proteinase-3, as well as matrix metalloproteinase (MMP-8 and MMP-9), which may contribute to alveolar destruction [19]. On the contrary, the number of pulmonary CD8^+^ T cells increases during higher stages of airflow limitation and emphysema [13], during which phase they release proteolytic enzymes which cause structural cell death via apoptosis and/or necrosis [20]. In contrast, lungs of stable COPD patients are populated by CD4 ^+^ Th1 and Th17 cells, which produce IFN-γ and IL-17A and IL-17F, respectively. The latter promote neutrophil accumulation at the site of injury increasing the release of granulocyte growth factors (G-CSF, GM-CSF) by epithelial cells [21]. It is worthy to point at the role of epithelial cells in this context, which can not only produce inflammatory mediators during COPD exacerbation, but also up-regulate their membrane expression of epithelial growth factor receptors (EGFR) responsible of metaplasia and increased risk of cancer [8].

### COPD and Cigarette Smoke: role of the oxidative stress

**Figure 2:**
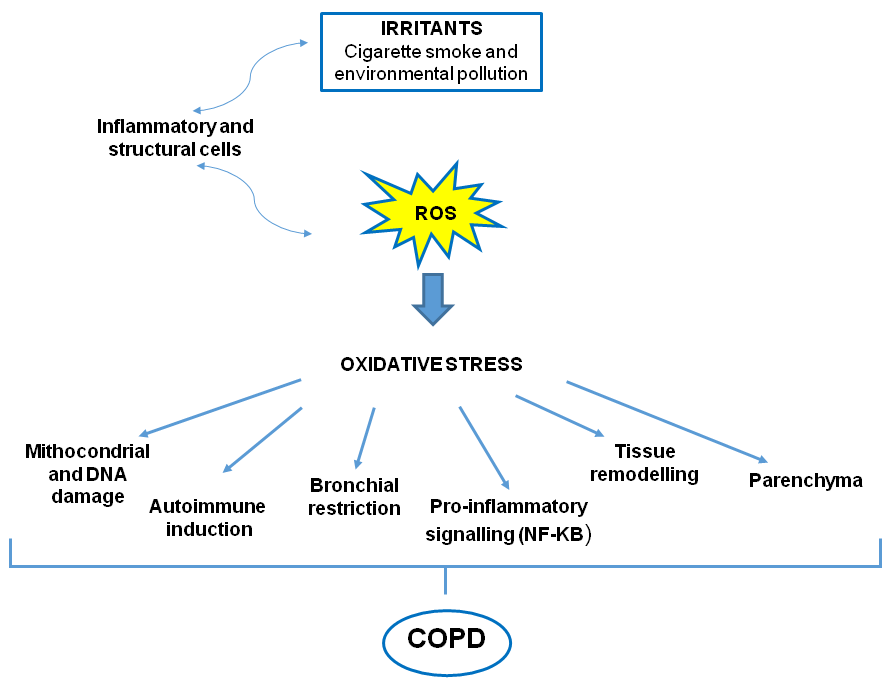
Oxidative stress in COPD Both oxidants generated from inhaled oxidants (cigarette smoke) and inflammatory cells in the lungs contribute to a burden of ROS, which drives many features of COPD.

Oxidative stress is considered a critical feature and a key mechanism in many molecular processes during the pathogenesis of COPD [22]. Patients have evidence of oxidative stress in the lungs, blood and skeletal muscle because mitochondrial dysfunctions lead to excessive production of reactive oxygen species (ROS) resulting in harmful effects, as damage to lipids, proteins and DNA [23] (Figure 2).

ROS in patients with COPD are produced by inflammatory (i.e. neutrophils, macrophages) and structural cells, (i.e. epithelial cells) activated into the airways. This event leads to alteration of the airways and parenchyma resulting into bronchoconstriction associated to the oxidation of the arachidonic acid and increase of inflammatory responses. Furthermore, oxidative stress triggers NF-κB and histone acetyltransferase activation, promoting the expression of multiple inflammatory genes, and down-regulation of anti-proteases, including α_1_-antitrypsin, resulting in acceleration of the breakdown of elastin in lung parenchyma [8].

As previously reported, CS is considered as the principal cause of COPD onset and it is known that exposure to CS increases levels of ROS [2, 24, 25]. Cigarettes contain about 10^15^ free radicals/puff, including reactive nitrogen and oxygen species (RNOS), which with endogenous RNOS produced by mitochondrial respiration, cause damage to the lungs, induce the release of pro-inflammatory cytokines and thus airway destruction, air trapping and lung hyperinflation [26]. Despite CS is a risk factor for COPD, only 15-20% of smokers develop COPD suggesting that genetic predisposition and environmental factors play an eligible role in the onset of this pathology [27].

### Inflammasome: components and activation in COPD pathogenesis

The characteristic of COPD is an altered immune response followed by chronic lung inflammation. Emerging scientific evidence suggests that persistent Nod-like Receptor 3 (NLRP3) inflammasome activation may be involved in the onset of COPD pathogenesis [9]. The inflammasome is a multimeric complex involved in caspase-1-dependent release of pro-inflammatory IL-1-like cytokines [28]. NLRP3 is an NLRs which contains a C-terminal leucine-rich repeat (LRR) domain, a central NACHT domain (or NBD: nucleotide-binding domain), and an N-terminal pyrin domain (PYR) [29]. NLRP3 inflammasome contains the adapter protein ASC (apoptosis speck-like protein), which has a caspase recruitment domain (CARD). ASC acts as a zipper, binding NLRP3 with pro-caspase 1, which in turn undergoes proteolytic cleavage that releases an active form of the enzyme, able to activate pro-IL-1β and pro-IL-18 into their active forms, promoting inflammation [2].

It is postulated that two signals are required for the canonical activation of NLRP3 inflammasome in mice [30], whereas a very recent paper from Dr. Hornung’s laboratory [31] discovered that the sole addition of lipopolysaccharide (LPS) to human monocytes is able to induce the activation of the inflammasome with the resultant release of IL-1β and IL-18 without cell death, as instead widely described [28]. The latter study imply that different levels exist for inflammasome activation between humans and mice. The first signal for inflammasome activation in murine cells involves the recognition of PAMPs or DAMPs by TLR4 or TLR2, TNF-receptor (TNFR) and IL-1 receptor (IL-1R) [28] that trigger NF-κB dependent gene expression. The second signal leads to the assembly of the components into the inflammasome structure and provides the intracellular recognition of DAMPs or PAMPs by NLRs which in turn bind to ASC leading to the recruitment and activation/autocleavage of caspase-1 and production of IL-1α, IL-1β, IL-33 and IL-18 which play a significant role in inflammatory processes [32]. Indeed, they facilitate many distinct systemic and localized responses, often acting as alarmins or dangers signals during inflammation and immune responses to pathogenic insults.

Several types of cellular stresses can trigger NLRP3 inflammasome activation. Increasing evidence indicates that chronic inflammation and immune responses in the onset and progression of COPD are correlated to chronic CS-exposure [9]. In particular, although still controversial on how ROS can directly or indirectly activate NLRP3, an early theory suggests that NADPH oxidase, the main source of bactericidal ROS in phagocytes, may be responsible for particle-induced inflammasome activation [30]. In support, recent evidence pinpoints at the role of mitochondria as the source of ROS involved in NLRP3 inflammasome activation [33-36]. Deregulation of mitochondrial function, resulting in increased mitochondrial ROS (mtROS) production, leads to NLRP3 inflammasome activation [37]. It is known that ROS can be toxic, but they also serve as signaling molecules that chemically modify cellular targets involved in inflammasome activation. It was recently demonstrated that inflammasome activators induced ROS-dependent association of thioredoxin (TRX)-interacting protein (TXNIP), a protein that during oxidative stress conditions binds NLRP3 and leads to its activation. In support, the genetic absence of TXNIP impairs NLRP3 inflammasome activation [38] due to the inability to trigger ROS [2]. In this context, CS exposure increases the levels of ROS that activate NLRP3 inflammasome [29, 39,40].

**Figure 3:**
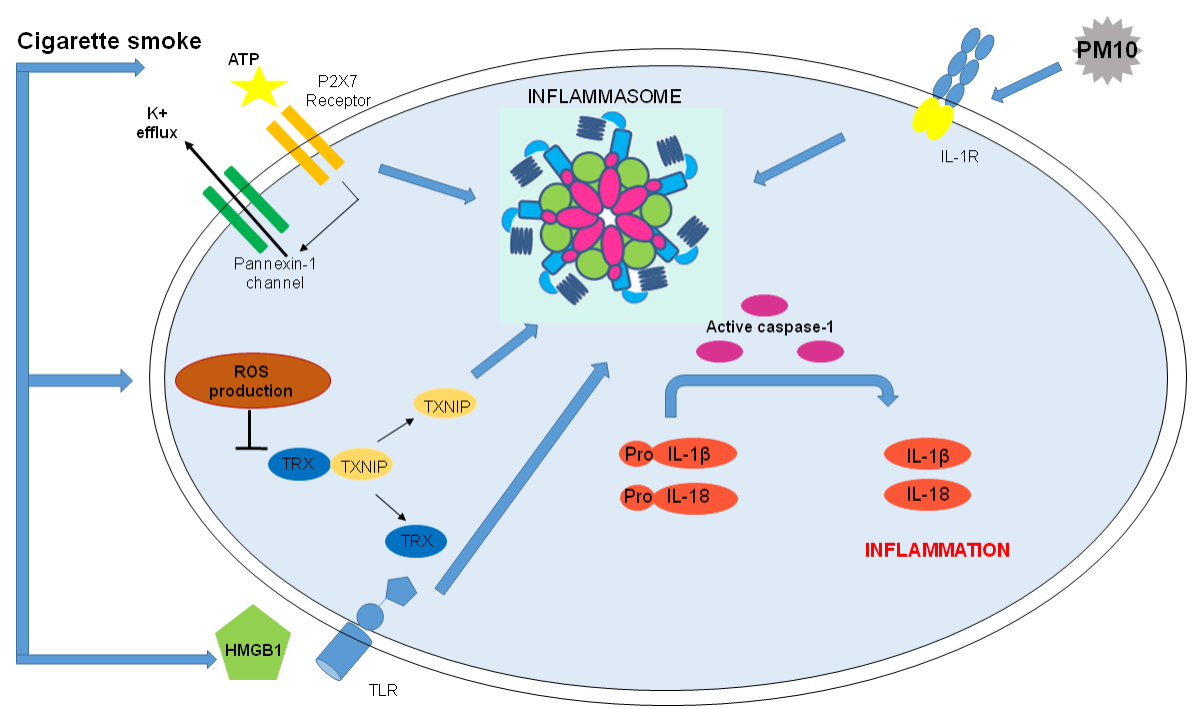
Correlation between COPD and Inflammasome activation Nowadays, the role of NLRP3 in COPD is not well defined, however there are different possible activators of the inflammasome in COPD onset and progression. Correlation between COPD and Inflammasome: ATP, linking P2X7 and altering cytosolic levels of ions, activates NLRP3 inflammasome. CS, major risk factor for COPD, increases ATP, an inflammasomal activator, in the BAL and bronchial tissue of COPD patients and it is also involved in CS-induced inflammation in mice. Moreover, CS increases levels of ROS which are involved in NLRP3 inflammasome activation and lead to HMGB1 secretion that, activating TLRs amplifies inflammasome-dependent response. Urban particulate matter (PM) is considered among NLRP3 inflammasome activators: PM10 activate NLRP3/IL-1receptor axis inducing inflammasome-dependent IL-1 like cytokine release.

Moreover, high levels of extracellular ATP (eATP) act as endogenous danger signal which triggers the activation of NLRP3 inflammasome via purino-receptor P2X7 binding [41]. The ligation of eATP to P2X7 alters cytosolic levels of ions. The intracellular decrease of K^+^ and increase of Ca^2+^ levels trigger NLRP3 activation [2]. As reported by Muller and collaborators [42], the lungs of CS-exposed mice show increased levels of eATP and ROS, due to P2X7 activation, resulting in NLRP3 assembly and activation in cells of bronchial tissues [2] (Figure 3). Thus, growing, although non-direct, evidence suggest that eATP and P2X7 play a key role in the development of COPD [43], as potentially confirmed by high levels of eATP in the BAL of patients with COPD [44].

Another risk factor for COPD is the exposure to urban particulate matter (PM), which is among inflammasome activators in airway epithelia [45] and immune cells [40]. Nowadays, the precise mechanism/s PM primes and activates NLRP3 inflammasome is not fully elucidated, because PM is a mixture of solid or liquid particles including sores, endotoxin and metals suspended in the air [29]. PM is deposited at different levels of the respiratory tract, depending on its size: coarse particles (PM_10_) in upper airways and fine particles (PM_2.5_) can accumulate in the lung parenchyma, inducing several respiratory diseases [46]. However, few studies have valued the effects of PM on NLRP3 inflammasome. It is demonstrated that PM_10_ activates NLRP3/IL-1 receptor axis (Figure 3) involving IL-1β, CCL-20 and GM-CSF production which are also linked to DC activation and neutrophilia [45].

Another inflammasome-dependent effect is the caspase-1 dependent-pyroptosis, a type of cell death characterized both by apoptosis and necrosis, resulting in the release of inflammatory mediators including IL-1α, IL-1β, IL-18 and HMGB1 (high mobility group box 1 protein) [29]. HMGB1, which inhibits macrophage phagocytosis of apoptotic cells [31], is highly detected in both airways and peripheral blood of COPD patients, negatively correlated to patients’ lung function [47]. It is known that acute and inflammatory responses in airway are associated with the accumulation of immune and structural cells undergoing apoptosis which need to be engulfed by phagocytes like macrophages and airway epithelial cells [48]. Alterations of this process leads to secondary necrosis of accumulating apoptotic cells, release of necrotic debris and inflammation amplification. Apoptotic epithelial, endothelial and immune cells are often observed in the lung of COPD patients. Therefore, it is likely that there is a defect in the uptake of apoptotic bronchial epithelial cells and neutrophils by macrophages in COPD patients, who currently smoke compared to non-smokers and healthy subjects [49]. However, the precise mechanism/s and biological relevance of pyroptosis require further investigation to define its role in inflammasome-mediated chronic inflammation in COPD.

In this scenario, the persistent activation of NLRP3, as well as its overexpression into the recruited and in tissue-resident cells, may promote chronic diseases [9].

### Role of the inflammasome in animal models of COPD

Several experimental pre-clinical approaches have been used to mimic the hallmark features of COPD. However, two main animal models are currently used:

1. Elastase emphysema model, which consists of a single dose of intranasal elastase instillation in female C57BL/6 mice [50]. This experimental procedure promotes a rapid (10 days) lung damage, which does not entirely translate what happens in humans, although it could be used to mimic acute exacerbations [51];

2. CS exposure model which can be distinguished in whole body or nose-only exposure of BALB/c mice [52]. The sole nose exposure to CS has the advantage to favor the development of COPD features such as pulmonary inflammation, mucus hypersecretion, airway remodelling and fibrosis, emphysema and impaired lung function, better mimicking the inhalation profile of human smokers compared to whole body exposure [53]. In particular, short-term mouse model of CS-induced COPD, set up by Beckett et al. [52], allows to observe COPD morphological features, as well as steroid unresponsiveness, after only 8 weeks of CS exposure. In particular, BALB/c mice are exposed to CS (12 cigarettes) twice per day (equivalent to a pack-a-day smoker) and 5 times per week for 1 to 12 weeks by using a smoke-exposure system.

Experimental inhalation of tobacco (whole body exposure to CS) shows that the inhibition of caspase-1 significantly decreases airway inflammation [54], implying the involvement of the inflammasome in COPD pathogenesis. Particular emphasis has been put on the role of NLRP3, which genetic absence reduces lower respiratory resistance, COPD index, compared to wild type controls [55]. NLRP3 knock-out mice exposed to CS show decreased activation of caspase-1 and subsequent lower release of IL-1β/IL-18 and neutrophil influx in the BAL [56, 45] (Figure 3). A very interesting study showed that ASC specks are highly released into the BAL of CS-induced COPD mouse model [57], amplifying the inflammatory response in an inflammasome-dependent manner. Indeed, the overexpression of IL-1β and IL-1α [58] in lung epithelium display a phenotype similar to COPD consisting of lung inflammation, emphysema and pulmonary fibrosis, further demonstrated by a reduced airway neutrophilia in response to CS in mice lacking IL-1R [13]. Interestingly, CS-induced pulmonary inflammation was dependent on IL-1RI and could be significantly attenuated by neutralizing IL-1α or IL-1β [58]. To date, pulmonary macrophages (AMs), DCs and lung epithelial cells express high levels of NLRP3 mRNA [59], which in response to specific stimuli (i.e. CS) can be induced [60]. In support, in a mouse model of cigarette smoke-driven acute COPD, airway neutrophilia was dependent on the release of IL-1β/IL-18 - in a NLRP3/ASC and caspase 1/11-dependent manner; whereas IL-1α appeared to involve caspase 1/11 independently of IPAF, AIM2 and NLRP3 [56-61], implying other inflammasome-dependent and –independent mechanism/s.

IL-18, another cytokine processed via NLRP3 inflammasome activation, appears to be linked as its over-expression via the induction of Caspase-1/11 by CS in murine lungs results in pulmonary inflammation, emphysematous lesions and airway remodeling [62-63]. In support, IL-18Rα knock-out mice do not show inflammation and emphysema compared to wild type mice and they are partially protected from CS-induced lung injury [63-64]. Furthermore, IL-18 expression in CS-exposed mice is associated with CD4^+^/CD8^+^/CD19^+^/NK1^1+^ cells, which contribute to COPD-like phenotype [62].

### Role of the inflammasome in stable COPD patients

High levels of IL-1β are found in the lungs of patients with COPD after CS exposure [65]. Interestingly, NLRP3 is over-expressed in the lung of stable COPD patients rather than non-smokers and smokers with normal spirometry, implying the correlation of NLRP3 mRNA expression to the severity of airflow obstruction [66]. However, the authors [66] did not observe caspase-1 activation (cleavage in the active p10 form) in both human lung tissue and PBMCs obtained from stable COPD patients, suggesting that the high levels of IL-1β and IL-18 were driven by a different mechanism. In sharp contrast, Franklin et al., showed that BAL fluid and PBMCs from COPD patients were rich of ASC specks, also accumulated in the lung, implying the activation of the caspase-1-dependent inflammasome, responsible of IL-1β release [57]. Importantly, in this latter study, macrophages obtained from stable COPD patients phagocyte BAL-derived ASC specks triggering the secretion of IL-1β without the induction of NLRP3 assembly [57]. This evidence could explain the discrepancies about the role of NLRP3 or not in COPD onset, pointing the attention at the experimental conditions that could be pivotal for the identification of the inflammasome activation. Another evidence that prove the involvement of the inflammasome in COPD is that higher levels of the active form of caspase-1 are observed in lung tissues of smokers, COPD and emphysema patients compared to non-smokers [67]. It was demonstrated that lung mRNA and protein levels of caspase-1 were similar between smokers, non-smokers and COPD patients, however sputum concentrations of the active caspase-1 were significantly higher in COPD patients [66]. In contrast, Di Stefano et al., found no correlation between NLRP3, caspase-1 and IL-1β responses when comparing stable COPD patients with smokers [68]. Instead, we found that the stimulation of smoker-derived PBMCs with ultrafine particles released higher IL-1-like cytokines in a NLRP3-dependent manner. Although the PBMCs were not from COPD patients, we demonstrated that the two main risk factors for COPD, smoking and air pollution, are able to induce NLRP3 inflammasome activation, most likely leading to the chronic inflammatory responses typical of COPD [40].

Moreover, IL-18 levels, as well as IL-1R-expression on CD8^+^ T lymphocytes, are significantly higher in the sputum and peripheral blood of COPD patients than those in healthy smokers and non-smokers, suggesting IL-18/IL-1R system is involved in stable COPD [9, 69]. However, the concentration of IL-18 levels in the BAL are similar across COPD patients, smokers and non-smokers [68], although some discrepancies were observed in IL-18 mRNA transcripts between the subjects [66]. In contrast, some studies reported that IL-18 producing CD8^+^ T cells were increased in the lungs of COPD patients [64] and IL-18 was expressed in 80% of cells of bronchiolar and alveolar epithelium, including AMs [70].

Therefore, many controversies are still present in the field, most likely due to technical issues and/or nature of COPD-derived biological samples. To note, though, stable COPD patients are under corticosteroid treatment, which should, unless unresponsiveness is registered, reduce IL-1β activity due to higher induction of IL-1RA [71].

### Role of the inflammasome in COPD exacerbation

COPD exacerbation, as reported by ERS guidelines, is characterized by shortness of breathlessness, dyspnea, cough and sputum production, and increased sputum purulence, with a negative impact on the quality of life of patients with COPD and promoting disease progression, hospital admissions and death. These symptoms are dependent on pro-inflammatory mediators, i.e. bradykinin, which receptor B1R is up-regulated and induced by IL-1β and LPS [72], implying the potential activation of the inflammasome. Indeed, Di Stefano et al,, suggested that the multimeric complex may be critical in COPD exacerbation [68], but not in stable COPD, as suggested by the inefficiency of Canakinumab, IL-1β blocking antibody, in stable patients [22] (Table I). It is likely that the role of inflammasome in exacerbated COPD patients is due to the higher release of ATP, oxidative stress and infections. In this regard, another issue needs to be considered, lung microbiome. It is reported that stable COPD patients have 25% of bacteria, whereas unstable/exacerbated COPD patients present more than 50% of bacteria, explaining the higher infection rate that can lead to exacerbation. Among bacteria, H. influenza, S. pneumoniae, *Moraxella catarrhalis* are detected. Similarly, viral infections are around 10% in stable COPD patients compared to 30-60% in severe/exacerbated COPD patients. Therefore, it is likely that the recognition of these bacteria/viruses by PRRs may lead to the inflammasome activation with the ensuing overexpression and release of IL-1 β in the sputum and lung tissue of exacerbated COPD patients [13]. However, no direct clinical and experimental data is reported to show the involvement of the inflammasome in COPD exacerbation maybe due to the difficulty to recruit human biological samples and to the limitations of animal models to mimic what happens in humans during COPD exacerbation.

**Table 1:** Randomized clinical trials on COPD targeting inflammasome-related effectors.

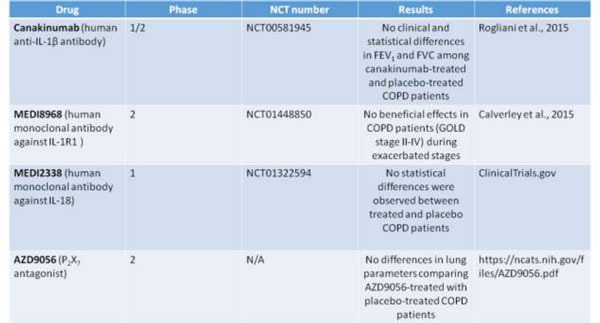

### Randomized clinical trials targeting inflammasome-dependent effectors

Despite the scientific progress, the biological mechanism/s underlying COPD pathogenesis is still unknown and, as reported by the guidelines of American Thoracic Society/European Respiratory Society, it still represents an urgent need to develop novel drugs, likely to inhibit COPD-associated chronic inflammation.

Some randomized clinical trials that target inflammasome-related effectors (i.e. IL-1α and IL-1β) have been performed on COPD patients considering moderate to severe stages (Table I).

As reported in ClinicalTrials.gov, from 2007 to 2011 Novartis launched a phase 1/2 study (NCT00581945; Randomized, Double-blind, Placebo Controlled, Exploratory Study) to evaluate the safety, tolerability and efficacy of multiple doses of Canakinumab , a human anti-IL-1β monoclonal antibody [73], versus placebo when administered via intravenous infusion. Pulmonary function of COPD patients was evaluated. Unfortunately the results of these clinical trials revealed no statistical differences in FEV_1_ (forced expiratory volume in one second) and FVC (forced vital capacity that is the total exhaled breath) among canakinumab-treated and placebo-treated COPD patients (Table I).

Another study involved MEDI8968, a human IgG2 monoclonal antibody against IL-1R1, able to bind both IL-1α and IL-1b, and injected intravenously (600mg) and subcutaneously (300 mg), did not show any beneficial effects in COPD patients (GOLD stage II-IV) during exacerbated stages (clinical trial: NCT01448850) [74].

Similarly, MEDI2338, a human IgG1 monoclonal antibody that binds IL-18, was studied for efficacy and safety in COPD. Again no statistical differences were observed between treated and placebo patients (clinical trial: NCT01322594)

Moreover, based on the potential involvement of eATP and P2X7 in COPD pathogenesis, during these last years the effect of AZD9056, a P2X7 antagonist which is able to bind the human P2X7 receptor with high selectivity and specificity and developed for the treatment of inflammatory conditions (i.e rheumatoid arthritis and Crohn’s disease), was studied in Phase 2 clinical trials in patients with moderate to severe COPD [75]. In this randomized study, patients with moderate to severe COPD received doses of 400 mg for 4 weeks. However, this study did not lead to positive results because lung parameters were not affected when comparing AZD9056-treated with placebo-treated COPD patients .

## CONCLUSIONS

This review points at the concept that chronic inflammation and immune responses play key roles in the development and progression of COPD. The references cited in this article evidence the involvement of macrophages, neutrophils, T-lymphocytes and DCs at orchestrating chronic inflammation in COPD onset. Among the cellular milieu, a molecular mechanism is highlighted: NLRP3 inflammasome activation and its ensuing products are highly present in the BAL, sputum and lung tissues of COPD patients. However, the exact mechanism needs to be further elucidated especially because not all the literature data reports a direct role of NLRP3 inflammasome, probably due to the difference between exacerbated and stable COPD patients, pointing at the role of corticosteroid treatment on the down-modulation of the inflammatory/immune response. To note, so far the sole NLRP3 inflammasome has been studied, although this does not rule out the role of other inflammasome-dependent and –independent receptors.

Here, human samples and experimental animal models suggest that NLRP3 inflammasome activation is involved in airway inflammation observed in COPD. It is known that cigarette smoke, risk factor and principal cause of COPD onset, is included among NLRP3 inflammasome activators via TLR-dependent and independent manner. Taken together, these data support that NLRP3 inflammasome may represent the link between inflammatory and immune responses in COPD pathogenesis. However, the exact mechanism underlying the inflammasome (not only NLRP3) activation in this disorder is still not clear. Therefore, further studies are needed to understand the role of this signaling complex in the development and progression of COPD.

More importantly, it is worth to point at the failure of phase II clinical trials in that the inhibition of IL-1α and IL-1β, as well as IL-18 ad P2X7, all underlying NLRP3-dependent and independent inflammasome, did not prove of beneficial activity in COPD patients. These data is of great relevance and suggest that although the presence of IL-1-like cytokines during exacerbation stages, other mechanisms are involved in COPD pathogenesis that most likely may lead to inflammasome activation. However, it is still important to note that moderate to severe COPD patients are under corticosteroid treatment especially during exacerbation phases. Therefore, it is of great relevance to understand the role of glucocorticoids in inflammasome-dependent signaling. It is well-known that corticosteroids inhibit IL-1-like cytokines (i.e. IL-1β). Therefore, in this context, a future clinical trial targeting inflammasome components and effectors needs to take into consideration the cross-talk between the two signaling pathways. Another consideration is that corticosteroids may down-regulate inflammasome components and thus effectors, probably explaining the inefficiency of the drugs in the randomized clinical trials (Table I). So far, it is not known whether corticosteroids inhibit inflammasome components and therefore further studies are needed to clarify the cross-talk between the two signaling pathways before programming future clinical trials.
